# The association between initial calculated driving pressure at the induction of general anesthesia and composite postoperative oxygen support

**DOI:** 10.1186/s12871-022-01959-0

**Published:** 2022-12-29

**Authors:** Koji Hosokawa, Katsuya Tanaka, Kayo Ishihara, Yukiko Yamazaki, Yuka Matsuki, Kenji Shigemi

**Affiliations:** 1 Department of Anesthesiology & Reanimatology, Faculty of Medical Sciences, University of Fukui, Fukui, Japan; 2 Department of Anesthesia, Fukui Prefectural Hospital, Fukui, Japan; 3 Faculty of Medical Sciences, University of Fukui, Fukui, Japan

**Keywords:** Respiratory function tests, Oxygen inhalation therapy, Respiratory physiology, Postoperative complication

## Abstract

**Purpose:**

Early discontinuation of postoperative oxygen support (POS) would partially depend on the innate pulmonary physics. We aimed to examine if the initial driving pressure (dP) at the induction of general anesthesia (GA) predicted POS prolongation.

**Methods:**

We conducted a single-center retrospective study using the facility's database. Consecutive subjects over 2 years were studied to determine the change in odds ratio (OR) for POS prolongation of different dP classes at GA induction. The dP (cmH_2_O) was calculated as the ratio of tidal volume (mL) over dynamic Crs (mL/cmH_2_O) regardless of the respiratory mode. The adjusted OR was calculated using the logistic regression model of multivariate analysis. Moreover, we performed a secondary subgroup analysis of age and the duration of GA.

**Results:**

We included 5,607 miscellaneous subjects. Old age, high scores of American Society of Anesthesiologist physical status, initial dP, and long GA duration were associated with prolonged POS. The dP at the induction of GA (7.78 [6.48, 9.45] in median [interquartile range]) was categorized into five classes. With the dP group of 6.5–8.3 cmH_2_O as the reference, high dPs of 10.3–13 cmH_2_O and ≥ 13 cmH_2_O were associated with significant prolongation of POS (adjusted OR, 1.62 [1.19, 2.20], *p* = 0.002 and 1.92 [1.20, 3.05], *p* = 0.006, respectively). The subgroup analysis revealed that the OR for prolonged POS of high dPs disappeared in the aged and ≥ 6 h anesthesia time subgroup.

**Conclusions:**

High initial dPs ≥ 10 cmH_2_O at GA induction predicted longer POS than those of approximately 7 cmH_2_O. High initial dPs were, however, a secondary factor for prolongation of postoperative hypoxemia in old age and prolonged surgery.

**Supplementary Information:**

The online version contains supplementary material available at 10.1186/s12871-022-01959-0.

## Introduction

High driving pressure (dP) is a strong predictor of mortality in acute respiratory distress syndrome (ARDS) [1]. The excessive dP generally increases the risk of ventilator-induced lung injury in ARDS [2]. For surgical subjects, inadequate respiratory strategies may increase the risk of postoperative pneumonia or atelectasis [3, 4]. A meta-analysis of studies in surgical subjects demonstrated that the lung-protective ventilatory strategy, including low tidal volume and/or PEEP during anesthesia, reduced the risk of postoperative pulmonary complication (PPC) [5, 6]. Recently, the incidence of PPCs reportedly increased with increasing dP during anesthesia with an individual PEEP management strategy [7–9]. The dP is one of the most important and clinically relevant measures of subject respiratory status. However, the dP immediately following the induction of general anesthesia (GA) have not been considered while the high dP forces more tight control of respiration during surgery.

Moreover, to measure the effect of intraoperative respiratory management, previous researchers have compared the rate of PPCs. The PPCs included various conditions, such as atelectasis, pneumonia, acute respiratory distress syndrome, pulmonary embolism, and other respiratory failures [5, 6, 10, 11]. As an alternative to PPC, we conceived that composite postoperative oxygen support (POS) duration may represent another clinically relevant outcome. Ventilator-associated events were defined as increases in inspiratory oxygen concentration for two consecutive days. These are used for surveillance as an alternative to ventilator-associated pneumonia [12, 13]. Considering this, we proposed that the duration of composite postoperative oxygen support (POS) is a possible surrogate marker of postoperative respiratory deterioration or hypoxemia [14]. The POS can be surveyed on a large scale under uniform criteria.

To solve the aforementioned unaddressed issues, we aimed to determine whether driving pressure immediately following the induction of GA predicted the need for POS. Moreover, we attempted to identify subgroups of subjects who required prolonged POS whose risk was predicted by the dP after induction of GA.

## Methods

We conducted a retrospective observational clinical study using the local electronic medical records, together with the anesthesia chart database. Ethical approval for this study was provided by the Research Ethics Committee of the Fukui University (#20210023). The requirement for written informed consent directly to the participants was waived by the Research Ethics Committee of the Fukui University because of the retrospective nature of the study. However, as opt-out policy, the information of study was cited on the hospital web site and the participants were allowed to deny the inclusion to the study via a direct contact to the researchers.

### Subjects and database

We included consecutive surgical subjects who received GA under mechanical ventilation from the anesthesia chart system, naming GAIA (Nihon Koden, Tokyo, Japan). The surgery date was from January 1, 2019, to December 31, 2020. The data of duplicated subjects who underwent sequential surgeries during a single hospital admission were deleted (Supplemental Fig. 1). We did not calculate the sample size required.

The datasets included the age; height; weight; American Society of Anesthesiologist (ASA) physical status classification; surgical categories; and the time of beginning and stop times of anesthesia, the beginning and stop times of surgery, the beginning and stop times of laparotomy, and the beginning and stop times of one-lung ventilation. Using the unique identity number for a single surgery, we collected the respiratory parameters measured on Aisys CS 2 (GE Healthcare, Chicago, IL), including the respiratory system compliance (Crs), tidal volume, peak pressure, positive end-expiratory pressure, during every minute of anesthesia. Moreover, we electronically collected the following data from the hospital medical records operated by IBM (Tokyo, Japan): admission date, discharge date, intensive care unit (ICU) admission date, ICU discharge date, the beginning date of mechanical ventilation, including non-invasive positive ventilation, the final date of mechanical ventilation, the beginning date of oxygen therapy, the final date of oxygen therapy, and survival state at hospital discharge. The aforementioned data were fed into the database.

### Outcomes

The odds ratio (OR) for prolonged POS in different dPs at GA induction was the primary outcome. Secondary outcomes included the length of POS, duration of postoperative mechanical ventilation, duration of postoperative ICU stay, and hospital mortality rate at different dPs.

### Respiratory parameters

The Crs was displayed on Aisys CS 2 under manufacture-driven calculation [in case of pressure control ventilation, the value of dynamic Crs used; Crs = tidal volume/(maximum pressure‒positive end expiratory pressure)]. Moreover, it measured the expiratory tidal volume. These values were stored on the anesthesia chart system and extracted every minute during anesthesia. Subsequently, the dP (cmH_2_O) was calculated as the ratio of tidal volume (mL) over Crs (mL/cmH_2_O). The dP following intubation denoted the mean of consecutive 9-min stored values post-intubation.

### Respiratory management

Respiratory management strategy was determined by attending anesthesiologists under the measurements of capnography and respiratory parameters displayed on Aisys CS 2. The tidal volume was limited below 8 ‒ 10 mL/ideal body weight. The use of zero end-expiratory pressure (ZEEP) was limited to brief periods during hepatectomy or other situations deemed necessary by anesthesiologists, and initial PEEP at the induction of GA was 5 cmH_2_O and decided to be increased in case of Crs decrease under the observation of Crs trends visualized on the electric anesthesia chart. The pressure-controlled ventilation with volume guarantee (PCV-VG) was ruled to be used mainly. Crs was displayed on Aisys CS 2 under manufacture-driven calculation, and for the measurement of Crs the plateau pressure was not required. Recruit maneuver was allowed anytime. We did not have protocolized ventilator management tools to minimize dP during anesthesia.

### Postoperative oxygen support

The composite POS included the use of mechanical ventilation, a nasal cannula, or face mask. The duration of POS was defined as one between the day of surgery to the final day on which the oxygen therapy was discontinued for 2 consecutive days. The administration of oxygen was determined by the ward staff or surgical physicians, mainly based on pulse oximetry values without concrete written criteria. We neither determined the protocols for its discontinuation nor implemented anesthesiologist-driven assessment tools for POS. Most surgical subjects received oxygen therapy for several hours following surgery or overnight until the next day of surgery for post-anesthetic concerns; therefore, ≥ 3 days of POS (i.e. later than post-operative day 2) was defined as prolonged POS.

### Statistical analysis

We divided the dP at the induction of GA into five categories using Jenks natural breaks classification method. We compared the duration of POS among these five categories using the Wilcoxon test or Kruskal–Wallis test. Kaplan Meier curves were separately plotted among the five categories. We performed a log rank test for analyzing the differences among the Kaplan Meier curves. Furthermore, we compared the adjusted OR of prolonged POS in different dPs with the reference category covering the median of dPs by implementing the logistic regression model to binary values. For numerical data, we used the Cox regression hazard model to calculate the hazard ratio (HR). We performed a multivariate analysis to adjust for the clinically relevant parameters, such as age, ASA physical status, categories of surgery, and the duration of anesthesia. We performed a subgroup analysis for the surgical category, age, ASA physical status, surgical schedule, duration of anesthesia, and other subject status. The combined database was managed, including cleanings on Microsoft Excel (Microsoft, Redmond, WA). All graphics and statistics were performed using Microsoft Excel or JMP 16 (SAS, Cary NC).

## Results

Of the 5,607 included subjects, 91.1% underwent an elective surgery (Table 1). PCV-VG was the respiratory mode in most cases (94.3%; Table 1) and the respiratory parameters at the induction of GA was shown in Supplemental Table 1.

**Table 1:** Patient characteristics

	All cases (*n* = 5,607)
Age (years)	65 [48, 74]
Male: Female	2,774 (50.5%): 2,833 (49.5%)
Body mass index (kg∙m^−2^)	22.8 [20.5, 25.6]
ASA physical status	
1, 1E	636 (11.3%), 76 (1.4%)
2, 2E	3,255 (58.1%), 230 (4.1%)
3, 3E	1,213 (21.6%), 191 (3.4%)
≥ 4, 4E	0 (0%), 3 (0.1%)
Emergent hospital admission	829 (14.8%)
Elective surgery	5,104 (91.1%)
Surgical category	
Cerebral	167 (3.0%)
Head or neck	1,343 (24.0%)
Chest	406 (7.2%)
Cardiovascular	176 (3.1%)
Upper abdomen	579 (10.3%)
Lower abdomen	1,233 (22.0%)
Extremity	849 (15.1%)
Spine	367 (6.6%)
Surface or wall	411 (7.3%)
Other	72 (1.3%)
Duration (min)	
Anesthesia	264 [186, 396]
Mechanical ventilation in the operation room	232 [156, 345]
Surgery	201 [81, 214]
Laparoscopic procedure	1,007 (18.0%)
Duration (min)	120 [71, 210]
One lung ventilation	399 (7.1%)

Values are presented as percentages or medians [interquartile ranges]

*ASA* American Society of Anesthesiologists

### Prolonged postoperative oxygen support and driving pressure

The POS was conducted for 1 day or 2 days in most cases (4,777 subjects; 85.2%) and was classified as normal (< 3 days) or prolonged (≥ 3 days). In the prolonged POS group, the duration of surgery was longer than in others (311 [170, 520] min vs. 140 [82, 236] min, *p* < 0.001; Supplemental Table 2). Furthermore, the prolonged POS group demonstrated longer hospital stay (24 [17, 37] days vs. 11 [7, 19] days, *p* < 0.001) and higher mortality (1.9% vs. 0.1%, *p* < 0.001) than others.

In the prolonged POS group, the dP at GA induction was higher than in the others (8.33 [6.80, 10.04] cmH_2_O vs. 7.71 [6.45, 9.34] cmH_2_O; Supplemental Table 2). The dP values correlated with the duration of POS as follows: POS (day) = 1.17 + 0.13 × dP (cmH_2_O) (F value, 55.17; *p* < 0.0001).

### Different driving pressure categories and outcomes

**Fig. 1:**
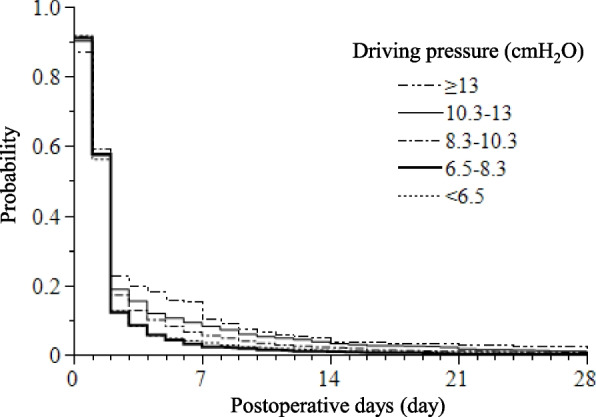
The probability of duration of postoperative oxygen support in different driving pressure categories. The duration of composite postoperative oxygen support has been compared among subjects divided into five classes according to the driving pressure (dP). Kaplan Meier curves demonstrate significant differences among the groups (*p* < 0.0001, log rank test)

**Fig. 2:**
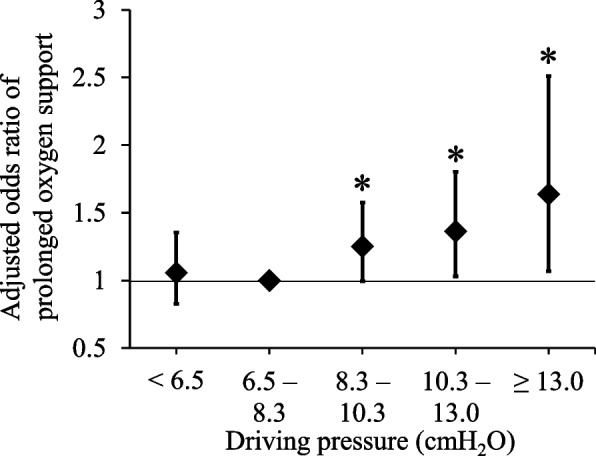
Prolonged postoperative oxygen support in different driving pressure categories. Subjects have been divided into five categories according to the driving pressure (dP). Considering a reference dP of 6.5–8.3 cmH_2_O, the three high dP groups show significantly high adjusted odds ratio for prolonged oxygen support

The subjects were categorized into five classes according to dPs as follows: < 6.5 cmH_2_O, 6.5–8.3 cmH_2_O, 8.3–10.3 cmH_2_O, 10.3–13.0 cmH_2_O, and ≥ 13.0 cmH_2_O (Table 2, Supplemental Table 3). The median duration of POS in all classes was 2 [1, 2] days (differences among classes; χ^2^, 10.05; *p* = 0.040, Kruskal–Wallis test; Table 2). The Kaplan Meier curve of POS varied among the groups (*p* < 0.0001, log rank test; Fig. 1). Considering the median dP was 7.7 cmH_2_O in the cohort, we set the reference as 6.5–8.3 cmH_2_O. Compared with the reference, the adjusted OR of prolonged POS gradually increased in the high dP groups of 8.3–10.3 cmH_2_O, 10.3–13 cmH_2_O, and ≥ 13 cmH_2_O (1.38 [1.8, 1.77], *p* = 0.011; 1.62 [1.19, 2.20], *p* = 0.002; and 1.92 [1.20, 3.05], *p* = 0.006, respectively; Fig. 2).

**Table 2:** Outcomes

	All cases (*n* = 5,607)	Driving pressure (cmH_2_O)					
		< 6.5 (*n* = 1,408)	6.5 ‒ 8.3 (*n* = 1,867)	8.3 ‒ 10.3 (*n* = 1,383)	10.3 ‒ 13.0 (*n* = 701)	≥ 13.0 (*n* = 209)	*p*-value
Postoperative oxygen support (day)	2 [1,2]	2 [1,2]	2 [1,2]	2 [1,2]	2 [1,2]	2 [1,2]	0.040
Postoperative duration of mechanical ventilation in applied cases (day)	4 [2,7]	4 [2,7]	3 [2,5]	4 [2,8]	6 [2,12]	4 [2,9]	0.056
Postoperative ICU stay in applied cases (day)	3 [2,7]	2 [2,5]	3 [2,5]	3 [2,7]	5 [2,9]	3 [2,8]	< 0.001
Hospital stay (day)	13 [8, 22]	12 [8, 21]	12 [8, 21]	14 [8, 22]	15 [8, 22]	15 [8, 25]	0.002
Hospital mortality	21 (0.37%)	0 (0%)	4 (0.21%)	9 (0.65%)	5 (0.71%)	3 (1.44%)	0.001

Values are presented as percentages or medians [interquartile ranges]

*ICU* intensive care unit

In subjects who required mechanical ventilation, dP did not affect the postoperative duration of mechanical ventilation (Table 2). In subjects who admitted to ICU, higher dP were associated with the longer postoperative ICU stay. Hospital stay and hospital mortality differed among different dP categories.

### Subgroup analysis

We examined the change in OR for prolonged POS among different dP categories in the subgroup analysis. In the non-elderly (< 80 years) subgroup, high dPs were associated with high OR for prolonged POS; however, this association was not seen in the aged (≥ 80 years) subgroup (Supplemental Fig. 1). Considering the ASA physical status, high dPs were associated with high OR for prolonged POS in the low ASA physical status (1, 2) subgroup, but not in the high ASA physical status (≥ 3) subgroup (Supplemental Fig. 1). Analyzing two classes of the duration of anesthesia (< 6 h and ≥ 6 h) demonstrated that high dPs were associated with high OR for prolonged POS in the < 6 h subgroup. Contrarily, the OR was similar in different dPs in the ≥ 6 h subgroup (Supplemental Fig. 1). Among different surgical categories, these associations disappeared, except for lower abdominal surgery (Supplemental Table 4).

## Discussion

Prolonged POS defined as ≥ 3 days following surgery was associated with advanced age, increased ASA physical status, and long duration of anesthesia. Initially high dP ≥ 10 cmH_2_O at GA induction was associated with an increased OR for prolonged POS. Since the association between high dPs and prolonged POS disappeared in the subgroups of advanced age, increased ASA physical status, or long duration of anesthesia, higher postintubation dPs was associated with prolonged POS in subjects without systemic comorbidities. Conversely, the initial dPs did not predict POS in subjects with advanced age, higher ASA physical status scores, or prolonged duration of surgery.

To measure postoperative respiratory deterioration or hypoxemia, we set the duration of POS as the primary outcome because it is easy to define and measure. In previous studies, PPC, including atelectasis or pulmonary infection, was the primary outcome [5, 6, 10, 11]. Since POS mainly indicated the extent of hypoxemia post-surgery, it would be a surrogate marker of PPCs. However, there are several indications for POS, including oxygen administration for purpose of preventing surgical site infection [15, 16]. To eliminate the influence of early phase of POS relating post-anesthesia routinely, we used the prolongation of POS as ≥ 3 days, since it suggests a post-operative respiratory complication. Then, the measurable values of the duration of POS were significantly associated with perioperative subject factors, including initial dP for ventilation.

Moderately high dP (≥ 10 cmH_2_O) at GA induction was significantly associated with an increased risk of POS, similar to the correlation between high plateau pressure and high risk of PPCs [17]. Ladha et al. categorized the subjects with the extent of plateau pressure into four classes based on their quartiles [17]. They set lowest quartile as the reference and demonstrated the OR. Contrarily, we used the Jenks natural breaks classification method that might maximize the difference among the groups. We believe this categorization is better, albeit needing a critical argument about how the dP break point values are categorized. Subsequently, the reference would be pointed on the median or the center of values. Recently, Zhang, et al., showed that PEEP optimization guided by minimum dP resulted in fewer PPCs than the fixed PEEP group [18]. Furthermore, low dP management during one-lung ventilation was related to the lower incidence of PPCs [19]. A large-scale post-hoc analysis of the LAS VEGAS study [11] showed that the time-weighted average dP was also associated with low rates of PPCs [9]. The researchers clearly showed that dPs were changed with the effects of the duration and types of surgery. We do not object to the importance of minimized dP strategy during the surgery. However, in the present study, we focused on initially high dP at GA induction considering that it suggests the low Crs in nature, indicating ventilation is difficult. Supposedly those subjects must be limited to low dP management during anesthesia. We did not show how the initial physiological status of surgical subjects influenced on the respiratory management duration the anesthesia. On this point, the present evidence has impact on further study seeking the best strategy for intraoperative ventilation.

The study protocol and concept had several limitations. First, owing to the retrospective observational design and the use of values stored on the database, we did not control for the respiratory settings using strict protocols. The target end-tidal CO_2_, pCO_2_, or the range of pressure and tidal volume were determined by bedside anesthetists supervised by anesthesiology specialists. Second, we assessed the calculated driving pressure derived from dynamic Crs. The plateau pressure under the volume control ventilation was not measured. Thus, the absolute value of the driving pressure may be misleading. Third, the single-minute values of Crs or tidal volume contained outliers. Fourth, the study population was diverse, and the main result was difficulty in utilization for a specific surgical patient. Fifth, we did not consider the results of the perioperative pulmonary function test, which is a possible gold standard for determining the respiratory function status. Sixth, importantly, POS lacked standard criteria for discontinuation. No articles have previously provided evidence for a correlation between the duration of oxygen therapy and the extent of postoperative pulmonary complications, despite the correlation being clinically natural. Moreover, we did not distinguish oxygen therapy without pulmonary disease (for example, for cardiac failure).

In summary, high dPs ≥ 10 cmH_2_O at GA induction predicted longer POS than dPs of 7 cmH_2_O. The high dP at GA induction would be helpful for predicting postoperative respiratory conditions in a limited group of young subjects who received short surgeries. Further study would clarify the difference of respiratory management between initially high dPs group and others and validate the clinical benefit of the presented prediction.

## Supplementary Information

**Additional file 1:** **Supplemental Figure 1.** Data collection process. Crs, respiratory system compliance; GA, general anesthesia; POS, postoperative oxygen support. **Supplemental Figure 2.** Subgroup analysis of prolonged postoperative oxygen support Subgroup analysis has been conducted for the age, American Society of Anesthesiologist (ASA) physical status, surgical schedule, and the duration of anesthesia. Subjects have been divided into five categories according to the driving pressure (dP). Considering a reference dP of 6.5–8.3 cmH_2_O, an association is observed between high dPs and high odds ratio for prolonged postoperative oxygen therapy in the young age group with low score of ASA physical status and short duration of anesthesia. **Supplemental Table 1.** Respiratory parameters at the induction of general anesthesia Values are presented as medians [interquartile ranges]. PCV-VG, pressure-controlled ventilation with volume guarantee. **Supplemental Table 2.** Prolonged postoperative oxygen support. Values are presented as medians [interquartile ranges]. ASA, American Society of Anesthesiologists; ICU, intensive care unit; PCV-VG, pressure-controlled ventilation with volume guarantee. **Supplemental Table 3.** Patient characteristics among the five driving pressure categories. Values are presented as percentages or medians [interquartile ranges]. ASA, American Society of Anesthesiologists. **Supplemental Table 4.** Subgroup analysis of prolonged postoperative oxygen support. Prolonged postoperative oxygen support was defined as ≥ 3 days of oxygen therapy after surgery. The Odds ratio was adjusted for age, American Society of Anesthesiologist physical status, and duration of anesthesia. * statistical significance. n.a., not applicable.

## Data Availability

The datasets generated and/or analyzed during the current study are not publicly available to avoid unintended use, but are available from the corresponding author on reasonable request.

## Abbreviations

ASA: American Society of Anesthesiologists
dP: Driving pressure
GA: General anesthesia
HR: Hazard ratio
ICU: Intensive care unit
OR: Odds ratio
POS: Postoperative oxygen support
PPC: Postoperative pulmonary complication
PCV-VG: Pressure-controlled ventilation with volume guarantee
Crs: Respiratory system compliance
ZEEP: Zero end-expiratory pressure
