# Supplementary material for: The association between initial calculated driving pressure at the induction of general anesthesia and composite postoperative oxygen support
Source: BMC Anesthesiol. 2022 Dec 29;22:411. doi: 10.1186/s12871-022-01959-0 (PMC9798593; doi:10.1186/s12871-022-01959-0)
Supplement: Supplementary file 1 — Additional file 1: Supplemental Figure 1. Data collection process. Crs, respiratory system compliance; GA, general anesthesia; POS, postoperative oxygen support. Supplemental Figure 2. Subgroup analysis of prolonged postoperative oxygen support Subgroup analysis has been conducted for the age, American Society of Anesthesiologist (ASA) physical status, surgical schedule, and the duration of anesthesia. Subjects have been divided into five categories according to the driving pressure (dP). Considering a reference dP of 6.5–8.3 cmH2O, an association is observed between high dPs and high odds ratio for prolonged postoperative oxygen therapy in the young age group with low score of ASA physical status and short duration of anesthesia. Supplemental Table 1. Respiratory parameters at the induction of general anesthesia Values are presented as medians [interquartile ranges]. PCV-VG, pressure-controlled ventilation with volume guarantee. Supplemental Table 2. Prolonged postoperative oxygen support. Values are presented as medians [interquartile ranges]. ASA, American Society of Anesthesiologists; ICU, intensive care unit; PCV-VG, pressure-controlled ventilation with volume guarantee. Supplemental Table 3. Patient characteristics among the five driving pressure categories. Values are presented as percentages or medians [interquartile ranges]. ASA, American Society of Anesthesiologists. Supplemental Table 4. Subgroup analysis of prolonged postoperative oxygen support. Prolonged postoperative oxygen support was defined as ≥ 3 days of oxygen therapy after surgery. The Odds ratio was adjusted for age, American Society of Anesthesiologist physical status, and duration of anesthesia. * statistical significance. n.a., not applicable. [file 12871_2022_1959_MOESM1_ESM.docx]

Supplemental Tables and Figures

The association between driving pressure at the induction of general anesthesia and duration of composite postoperative oxygen support

Koji Hosokawa, MD, PhD*, Katsuya Tanaka, MD, Kayo Ishihara, MS,

Yukiko Yamazaki, MD, Yuka Matsuki, MD, PhD, Kenji Shigemi, MD, PhD

Supplemental Table 1. Respiratory parameters at the induction of general anesthesia

|  | All cases (n=5,607) |
| --- | --- |
|  |  |
| Tidal volume per body weight (mL/kg) | 6.8 [6.2, 7.5] |
| Tidal volume per predicted body weight (mL/kg) | 9.8 [8.6, 11.4] |
| Peak pressure (cmH_2_O) | 14 [13, 16] |
| Positive end-expiratory pressure (cmH_2_O) | 4.7 [4.1, 5.1] |
| Driving pressure (cmH_2_O) | 7.8 [6.5, 9.5] |
| Respiratory compliance (mL/cmH_2_O) | 52 [43, 63] |
| Mode of ventilation |  |
| PCV-VG | 5,267 (94.3%) |
| PCV | 167 (3.0%) |
| Other | 152 (2.7%) |

Values are presented as medians [interquartile ranges]. PCV-VG, pressure-controlled ventilation with volume guarantee.

Supplemental Table 2. Prolonged postoperative oxygen support.

|  | | | All cases (n=5,607) | Postoperative oxygen support | | | |
| --- | --- | --- | --- | --- | --- | --- | --- |
|  |  |  |  | <3 days (n=4,777) | ≥3 days (n=830) | p-value | |
| Age (years) | | | 65 [48, 74] | 63 [46, 73] | 71 [62, 79] | <.0001 | |
| Male: Female | | | 50.5%: 49.5% | 49.3%: 50.7% | 57.7%: 42.3% | <.0001 | |
| Body mass index (kg∙m^-2^) | | | 22.8 [20.5, 25.6] | 22.8 [20.5, 25.6] | 23.0 [20.4, 25.7] | 0.7158 | |
| ASA physical status | | |  |  |  | <.0001 | |
|  | 1, 1E | | 11.3%, 1.4% | 13.1%, 1.5% | 1.33%, 0.5% |  | |
|  | 2, 2E | | 58.1%, 4.1% | 61.5%, 3,7% | 38.6%, 6.3% |  | |
|  | 3, 3E | | 21.6%, 3.4% | 18.9%, 1.3% | 37.6%, 15.5% |  | |
|  | ≥4, 4E | | 0.0%, 0.1% | 0.0%, 0.0% | 0.0%, 0.2% |  | |
| Emergent hospital admission | | | 14.8% | 11.9% | 31.7% | <.0001 | |
| Elective surgery | | | 91.1% | 93.4% | 77.5% | <.0001 | |
| Surgical category | | |  |  |  | <.0001 | |
|  | Cerebral | | 3.0% | 2.8% | 4.0% |  | |
|  | Head or neck | | 24.0% | 27.2% | 5.3% |  | |
|  | Chest | | 7.2% | 7.3% | 6.9% |  | |
|  | Cardiovascular | | 3.1% | 0.9% | 15.9% |  | |
|  | Upper abdomen | | 10.3% | 7.6% | 26.0% |  | |
|  | Lower abdomen | | 22.0% | 20.1% | 32.8% |  | |
|  | Extremity | | 15.1% | 17.1% | 4.2% |  | |
|  | Spine | | 6.6% | 7.3% | 2.4% |  | |
|  | Surface or wall | | 7.3% | 8.3% | 2.1% |  | |
|  | Other | | 1.3% | 1.4% | 0.50% |  | |
| Duration (min) | | |  |  |  |  | |
|  | Anesthesia | | 264 [186, 396] | 244 [180, 354] | 426 [276, 650] | <.0001 | |
|  | Mechanical ventilation in the operation room | | 232 [156, 345] | 224 [148, 332] | 351 [222, 557] | <.0001 | |
|  | Surgery | | 201 [81, 214] | 140 [82, 236] | 311 [170, 520] | <.0001 | |
| Laparoscopic procedure | | | 18.0% | 18.3% | 16.3% | 0.1682 | |
|  | Duration (min) | | 120 [71, 210] | 118 [70, 207] | 154 [77, 252] | 0.0076 | |
| One lung ventilation | | | 7.1% | 7.1% | 7.1% | 0.9926 | |
| Respiratory parameter at the induction of general anesthesia | | |  |  |  |  | |
|  | Driving pressure (cmH_2_O) | | 7.78 [6.48, 9.45] | 7.71 [6.45, 9.34] | 8.33 [6.80, 10.04] | <.0001 | |
|  | Tidal volume per body weight (mL/kg) | | 6.8 [6.2, 7.5] | 6.9 [6.2, 7.5] | 6.8 [6.1, 8.1] | 0.0011 | |
|  | Respiratory compliance (mL/cmH_2_O) | | 52 [43, 63] | 53 [44, 63] | 48 [39, 61] | <.0001 | |
| Mode of ventilation | | |  |  |  | 0.3794 | |
|  | PCV-VG | | 94.3% | 94.5% | 93.3% |  | |
|  | PCV | | 3.0% | 2.9% | 3.3% |  | |
|  | Other | | 2.7% | 2.6% | 3.4% |  | |
| Postoperative oxygen therapy (day) | | | 2 [1, 2] | 2 [1, 2] | 5 [3, 9] | <.0001 |  |
| Mechanical ventilation 28 days after surgery | | | 5.7% | 0.7% | 34.6% | <.0001 |  |
|  | | Duration in applied cases (day) | 4 [2, 7] | 2 [2, 2] | 4 [3, 8] | <.0001 |  |
| Admission to ICU | | | 11.0% | 3.5% | 53.7% | <.0001 |  |
|  | | Postoperative ICU stay in applied cases (day) | 3 [2, 7] | 2 [2, 2] | 4 [2, 8] | <.0001 |  |
| Hospital stay (day) | | | 13 [8, 22] | 11 [7, 19] | 24 [17, 37] | <.0001 |  |
| Mortality | | | 0.4% | 0.1% | 1.9% | <.0001 |  |

Values are presented as medians [interquartile ranges]. ASA, American Society of Anesthesiologists; ICU, intensive care unit; PCV-VG, pressure-controlled ventilation with volume guarantee.

Supplemental Table 3. Patient characteristics among the five driving pressure categories.

|  | | Driving pressure (cmH_2_O) | | | | | |
| --- | --- | --- | --- | --- | --- | --- | --- |
|  |  | <6.5  (n=1,408) | 6.5 ‒ 8.3  (n=1,867) | 8.3 ‒ 10.3  (n=1,383) | 10.3 ‒ 13.0  (n=701) | ≥13.0  (n=209) | p-value |
| Age (years) | | 62 [46, 72] | 65 [47, 74] | 67 [51, 75] | 66 [47, 74] | 63 [44, 73] | <0.001 |
| Male: Female | | 54.0%: 46.0% | 50.6%: 49.4% | 50.0%: 50.0% | 45.7%: 54.4% | 45.5%: 54.6% | 0.004 |
| Body mass index (kg∙m^-2^) | | 21.0 [19.2, 22.9] | 22.6 [20.6, 25.4] | 24.2 [21.4, 26.8] | 25.1 [22.4, 28.2] | 23.5 [26.5, 31.1] | <0.001 |
| ASA- physical status ≥3 | | 244 (17%) | 407 (30%) | 415 (30%) | 234 (33%) | 99 (47%) | <0.001 |
| Surgical category | | | | | | | <0.001 |
|  | Chest | 4.9% | 6.1% | 9.1% | 9.6% | 13.4% |  |
|  | Upper abdomen | 12.0% | 9.6% | 10.6% | 8.8% | 9.6% |  |
|  | Lower abdomen | 25.2% | 23.9% | 19.5% | 16.8% | 18.7% |  |
|  | Other | 57.9% | 60.4% | 60.8% | 64.8% | 58.3% |  |
| Duration of surgery (min) | | 275 [192, 408] | 258 [182, 380] | 268 [190, 404] | 270 [189, 400] | 230 [173, 382] | 0.0249 |

Values are presented as percentages or medians [interquartile ranges]. ASA, American Society of Anesthesiologists.

Supplemental Table 4. Subgroup analysis of prolonged postoperative oxygen support

| Surgical category | |  | Odds ratio | | | | |
| --- | --- | --- | --- | --- | --- | --- | --- |
|  |  | n | Driving pressure (cmH_2_O) | | | | |
|  |  |  | <6.5 | 6.5 ‒ 8.3 | 8.3 ‒ 10.3 | 10.3 ‒ 13.0 | ≥13.0 |
|  | Cerebral | 167 | n.a. | Ref | n.a. | n.a. | n.a. |
|  | Head or neck | 1,343 | 1.12 [0.14, 8.53] | Ref | 0.52 [0.05, 5.94] | 2.06 [0.27, 15.86] | 2.97 [0.24, 15.86] |
|  | Chest | 406 | 2.18 [0.18, 26.78] | Ref | 0.87 [0.07, 12.12] | 2.82 [0.22, 36.06] | 2.11 [0.07. 60.10] |
|  | Cardiovascular | 176 | 0.76 [0.13, 4.37] | Ref | 1.82 [0.55, 6.01] | 3.33 [0.92, 11.72] | 1.82 [0.55, 6.01] |
|  | Upper abdomen | 579 | 6.79 [0.73, 63.16] | Ref | 3.24 [0.34, 30.84] | 8.78 [0.92, 83.35] | 7.25 [0.50, 105.14] |
|  | Lower abdomen | 1,233 | 1.01 [0.17, 6.68] | Ref | 1.71 [0.38, 7.71] | 1.81 [0.33, 9.93] | 6.23 [1.04, 37.20]* |
|  | Extremity | 849 | n.a. | Ref | n.a. | 0.60 [0.04, 9.57] | 5.40 [0.43, 67.45] |
|  | Spine | 367 | n.a. | Ref | n.a. | n.a. | n.a. |
|  | Surface or wall | 411 | n.a. | Ref | n.a. | 2.21 [0.08, 60.43] | n.a. |
|  | Other | 76 | n.a. | Ref | n.a. | n.a. | n.a. |

Prolonged postoperative oxygen support was defined as ≥ 3 days of oxygen therapy after surgery. The Odds ratio was adjusted for age, American Society of Anesthesiologist physical status, and duration of anesthesia. * statistical significance. n.a., not applicable.

Supplemental Figure 1. Data collection process


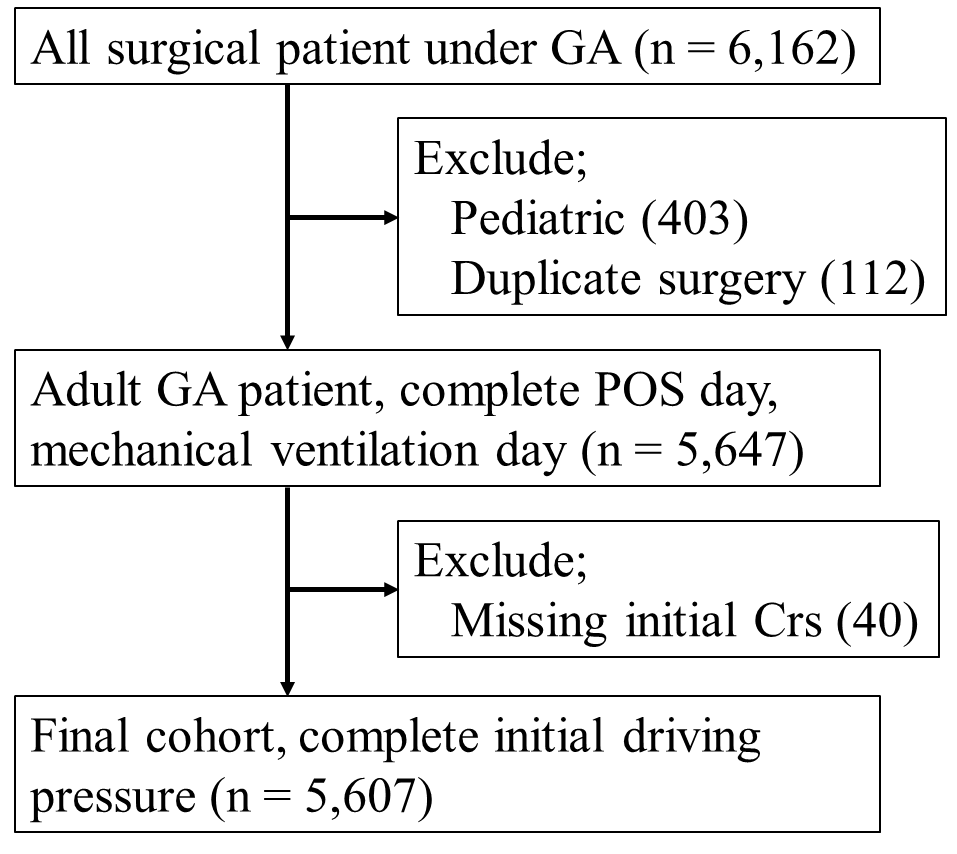


Crs, respiratory system compliance; GA, general anesthesia; POS, postoperative oxygen support.

Supplemental Figure 2. Subgroup analysis of prolonged postoperative oxygen support


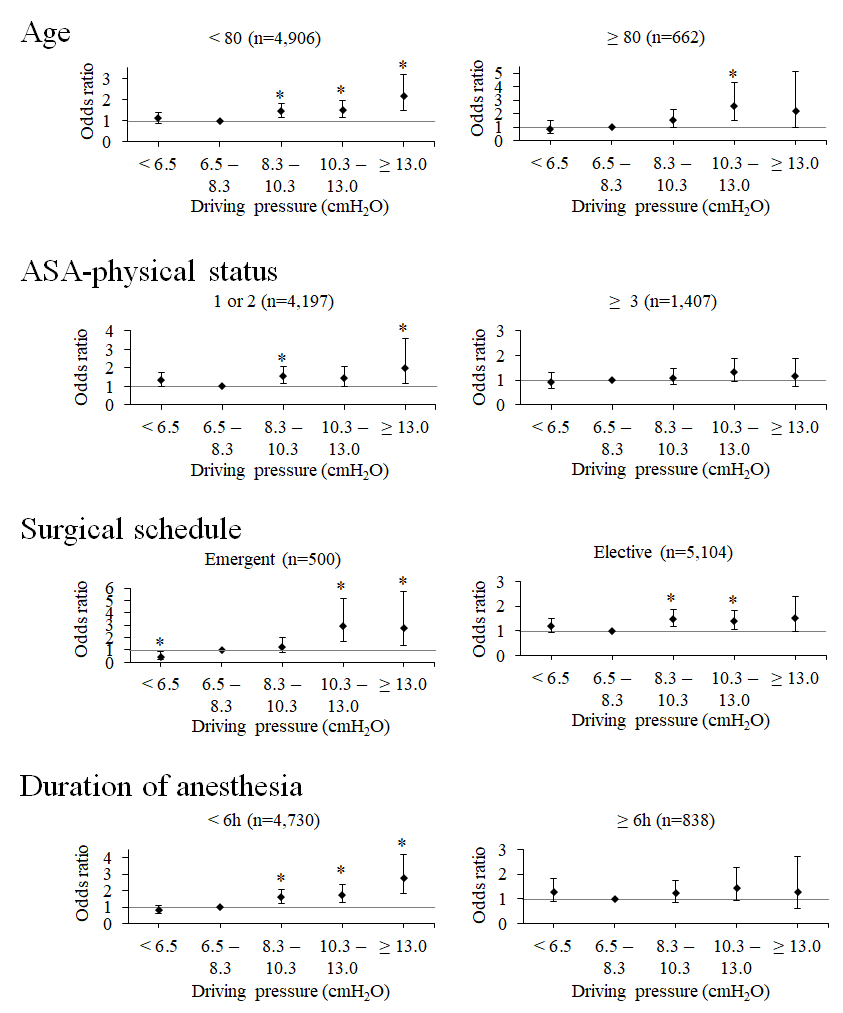


Subgroup analysis has been conducted for the age, American Society of Anesthesiologist (ASA) physical status, surgical schedule, and the duration of anesthesia. Subjects have been divided into five categories according to the driving pressure (dP). Considering a reference dP of 6.5–8.3 cmH_2_O, an association is observed between high dPs and high odds ratio for prolonged postoperative oxygen therapy in the young age group with low score of ASA physical status and short duration of anesthesia.
